# Benzylammonium hepta­noate–hepta­noic acid (1/1)

**DOI:** 10.1107/S1600536813003012

**Published:** 2013-02-06

**Authors:** Mary H. Wood, Stuart M. Clarke

**Affiliations:** a BP Institute and Department of Chemistry, University of Cambridge, Cambridge CB3 0EZ, England

## Abstract

The title salt, C_7_H_10_N^+^·C_7_H_13_O_2_^−^·C_7_H_14_O_2_, is an unusual 2:1 stoichiometric combination of two carb­oxy­lic acid mol­ecules and one amine. Although there are crystal structures of a number of 1:1 complexes reported in the literature, 2:1 acid amine complexes are rather uncommon. In this case, a proton is transferred between one acid mol­ecule and the amine to give an acid anion and an ammonium cation whilst the other carb­oxy­lic acid remains protonated. The species inter­act strongly *via* electrostatic forces and hydrogen bonds. In addition we note that the N atom of the ammonium group makes four close contacts to surrounding O atoms. Three of these are hydrogen bonds with neighbouring acid anions while the fourth does not involve a hydrogen atom but is directed towards the carbonyl O atom of the protonated acid. Each of the acid anion O atoms accepts two hydrogen bonds from adjacent N atoms. There is also evidence of short C—H⋯O contacts. There is disorder (occupancy ratio 0.51:0.49) in the alkyl chain of one of the carb­oxy­lic acid mol­ecules.

## Related literature

For spectroscopic studies of acid–amine complexes, see: Karlsson *et al.* (2000 ▶); Kohler *et al.* (1981 ▶); Smith *et al.* (2001 ▶, 2002 ▶); Klokkenburg *et al.* (2007 ▶). For recent diffraction studies of acid–amine complexes, see: Jefferson *et al.* (2011 ▶); Sun *et al.* (2011 ▶); Wood & Clarke (2012*a* ▶,*b* ▶).
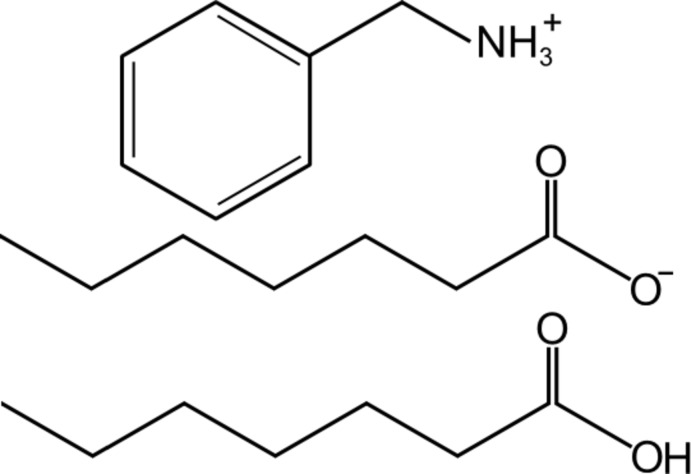


## Experimental

### 

#### Crystal data

C_7_H_10_N^+^·C_7_H_13_O_2_^−^·C_7_H_14_O_2_
*M* *_r_* = 367.52
Monoclinic, 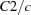

*a* = 25.5516 (5) Å
*b* = 6.3250 (1) Å
*c* = 27.9899 (6) Å
β = 90.639 (1)°
*V* = 4523.27 (15) Å^3^
*Z* = 8
Mo *K*α radiation
μ = 0.07 mm^−1^
*T* = 180 K
0.23 × 0.05 × 0.05 mm

#### Data collection

Nonius KappaCCD diffractometer
Absorption correction: multi-scan (*SORTAV*; Blessing, 1995 ▶) *T*_min_ = 0.910, *T*_max_ = 1.000
22723 measured reflections
5085 independent reflections
2353 reflections with *I* > 2σ(*I*)
*R*_int_ = 0.060

#### Refinement

*R*[*F* ^2^ > 2σ(*F* ^2^)] = 0.068
*wR*(*F* ^2^) = 0.238
*S* = 0.98
5085 reflections
232 parameters
10 restraints
H-atom parameters constrained
Δρ_max_ = 0.29 e Å^−3^
Δρ_min_ = −0.20 e Å^−3^

### 

Data collection: *COLLECT* (Nonius, 1998 ▶); cell refinement: *SCALEPACK* (Otwinowski & Minor, 1997 ▶); data reduction: *DENZO* (Otwinowski & Minor, 1997 ▶) and *SCALEPACK*; program(s) used to solve structure: *SIR92* (Altomare *et al.*, 1994 ▶); program(s) used to refine structure: *SHELXL97* (Sheldrick, 2008 ▶); molecular graphics: *Mercury* (Macrae *et al.*, 2008 ▶); software used to prepare material for publication: *SHELXL97*.

## Supplementary Material

Crystal structure: contains datablock(s) I, global. DOI: 10.1107/S1600536813003012/mw2101sup1.cif

Structure factors: contains datablock(s) I. DOI: 10.1107/S1600536813003012/mw2101Isup2.hkl

Supplementary material file. DOI: 10.1107/S1600536813003012/mw2101Isup3.cml

Additional supplementary materials:  crystallographic information; 3D view; checkCIF report

## Figures and Tables

**Table 1:** Hydrogen-bond geometry (Å, °)

*D*—H⋯*A*	*D*—H	H⋯*A*	*D*⋯*A*	*D*—H⋯*A*
N1—H1*A*⋯O1^i^	0.91	1.87	2.773 (3)	171
N1—H1*B*⋯O2^ii^	0.91	1.96	2.823 (3)	158
N1—H1*C*⋯O2	0.91	1.90	2.781 (3)	164
O3—H3⋯O1	0.84	1.78	2.610 (3)	172
C1—H1*D*⋯O4^iii^	0.99	2.60	3.542 (4)	160
C3—H3*A*⋯O2^i^	0.95	2.59	3.456 (4)	152

Symmetry codes: (i) 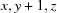; (ii) 
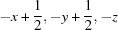
; (iii) 
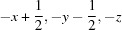
.

**Fig. 1.**
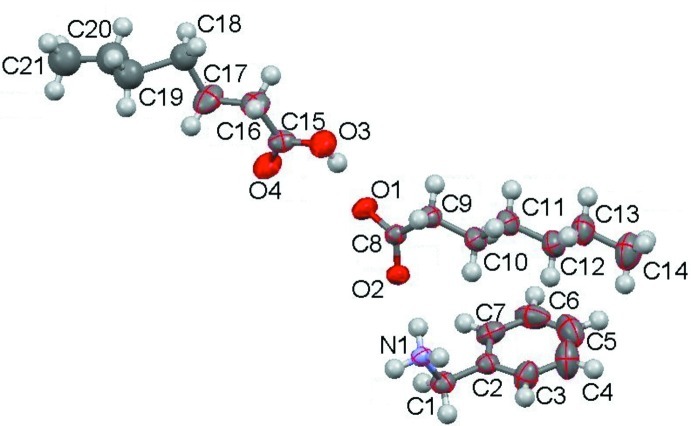
Illustration of the title compound with atom labels.

**Fig. 2.**
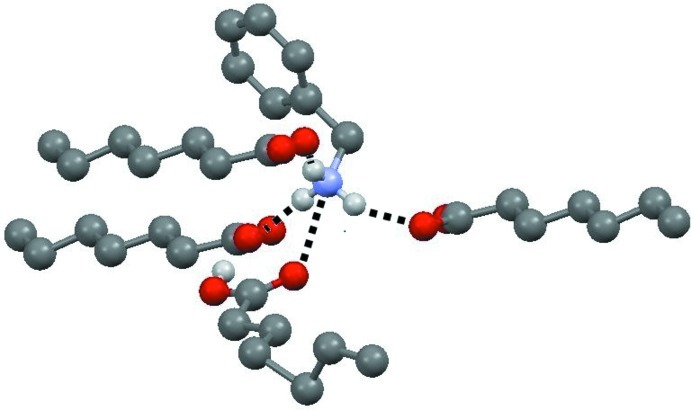
Three hydrogen bonds around each ammonium group nitrogen atom indicated by dotted black lines. The additional interaction of the carbonyl group of the protonated acid and the nitrogen atom of the ammonium ion is also indicated by a black dotted line.

**Fig. 3.**
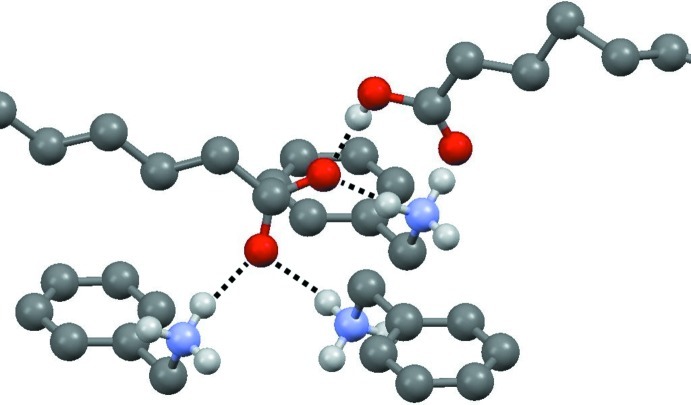
Hydrogen bonding around each carboxylate ion showing four hydrogen bonds, two for each oxygen, indicated by dotted black lines.

**Fig. 4.**
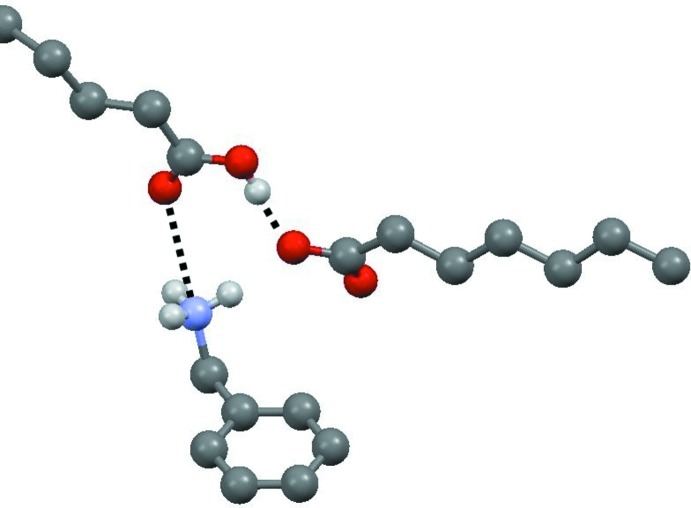
Illustration of the two non-covalent bonds made by the protonated acid molecules indicated by black dotted lines.

## supplementary crystallographic information

### Comment

Stable complexes formed between simple alkyl carboxylic acids and alkyl amines have been reported *e.g.* (Karlsson *et al.*, 2000; Wood *et al.*, 2012*a*; Wood *et al.*, 2012*b*) most commonly from spectroscopic studies. Interestingly there also exist examples of 2:1 and 3:1 acid amine complexes, usually in an acid-rich environment (Sun *et al.*, 2011; Kohler *et al.*, 1981). No equivalent amine-rich complexes have yet been observed, although (Smith *et al.*, 2001) and (Smith *et al.*, 2002) have reported a diamine complex formed between methylamine and dnsa due to deprotonation of the phenolic group in the acid (Smith *et al.*, 2002).

Such acid:amine complexes are generally considered to derive their stablity from the complete transfer of a proton from the acid to the amine with subsequent cation-anion electrostatic interaction and strong hydrogen-bond formation. In 2:1 or higher stoichiometry complexes, the hydrogen bond is considered to extend over the three (or more) species involved. However, because there are very limited single crystal diffraction data, the exact form of the interactions are not clear.

In this paper the crystal structure of the 2:1 complex formed by two heptanoic acid molecules and benzylamine is reported. This complex results from the donation of a proton from one acid to the base, forming a carboxylate anion and an ammonium cation. This pair of ions, along with an additional protonated acid molecule, forms the structure illustrated in Figure 1.

All these species interact strongly by electrostatic forces and hydrogen bonding. Figure 2 illustrates the non-covalent interactions around one ammonium ion. There are three hydrogen bonds around each ammonium group nitrogen atom (indicated by dotted black lines in the Figure). Unexpectedly, an additional short contact is observed between the carbonyl group of a protonated acid and the nitrogen atom of the ammonium ion with no hydrogen atom. This interaction is also indicated by a black dotted line in Figure 2.

Figure 3 illustrates the four hydrogen bonds around each carboxylate ion. Interestingly there are two hydrogen bonds evident for each oxygen (indicated by dotted black lines in the Figure).

An illustration of the two hydrogen bonds made by the protonated acid molecules is given in Figure 4. The non-protonated oxygen forms a hydrogen bond with an adjacent carboxylate ion. The other carbonyl group appears to interact with the nitrogen of an ammonium ion.

The alkyl chain of the protonated acid group shows a significant degree of conformational disorder. The chain is not all *trans* but shows *gauche* conformers.

### Experimental

Heptanoic acid and benzylamine, with purities of 99.8% and 99.7% respectively (titration and GC), were obtained from Sigma Aldrich and used without further purification. A small volume (approximately 1 mL) of each material was placed into two small vials and placed inside a larger vial with an inert atmosphere of nitrogen for a number of weeks, during which numerous crystals grew, particularly on a sample of polypropylene included as a nucleating surface, and around the top of the outer vial. Reaction of the amine with atmospheric CO_2_, was prevented by the inert atmosphere (Sun *et al.* 2011). The samples were measured at a temperature of 180 K.

### Crystal data

**Table:**

C_7_H_10_N^+^·C_7_H_13_O_2_^−^·C_7_H_14_O_2_	*F*(000) = 1616
*M**_r_* = 367.52	*D*_x_ = 1.079 Mg m^−^^3^
Monoclinic, *C*2/*c*	Mo *K*α radiation, λ = 0.71073 Å
Hall symbol: -C 2yc	Cell parameters from 15490 reflections
*a* = 25.5516 (5) Å	θ = 1.0–27.5°
*b* = 6.3250 (1) Å	µ = 0.07 mm^−^^1^
*c* = 27.9899 (6) Å	*T* = 180 K
β = 90.639 (1)°	Block, colourless
*V* = 4523.27 (15) Å^3^	0.23 × 0.05 × 0.05 mm
*Z* = 8	

### Data collection

**Table:**

Nonius KappaCCD diffractometer	2353 reflections with *I* > 2σ(*I*)
Radiation source: fine-focus sealed tube	*R*_int_ = 0.060
Thin slice ω and φ scans	θ_max_ = 27.5°, θ_min_ = 1.6°
Absorption correction: multi-scan (*SORTAV*; Blessing, 1995)	*h* = −32→32
*T*_min_ = 0.910, *T*_max_ = 1.000	*k* = −8→8
22723 measured reflections	*l* = −34→36
5085 independent reflections	

### Refinement

**Table:**

Refinement on *F*^2^	Primary atom site location: structure-invariant direct methods
Least-squares matrix: full	Secondary atom site location: difference Fourier map
*R*[*F*^2^ > 2σ(*F*^2^)] = 0.068	Hydrogen site location: inferred from neighbouring sites
*wR*(*F*^2^) = 0.238	H-atom parameters constrained
*S* = 0.98	*w* = 1/[σ^2^(*F*_o_^2^) + (0.1334*P*)^2^] where *P* = (*F*_o_^2^ + 2*F*_c_^2^)/3
5085 reflections	(Δ/σ)_max_ < 0.001
232 parameters	Δρ_max_ = 0.29 e Å^−^^3^
10 restraints	Δρ_min_ = −0.20 e Å^−^^3^

### Special details

**Table:**

Experimental. Part of the C_7_H_14_O_2_ molecule is disordered over two sites. In the refinement, the disordered carbon atoms were assigned common isotropic displacement parameters and geometric constraints.
Geometry. All e.s.d.'s (except the e.s.d. in the dihedral angle between two l.s. planes) are estimated using the full covariance matrix. The cell e.s.d.'s are taken into account individually in the estimation of e.s.d.'s in distances, angles and torsion angles; correlations between e.s.d.'s in cell parameters are only used when they are defined by crystal symmetry. An approximate (isotropic) treatment of cell e.s.d.'s is used for estimating e.s.d.'s involving l.s. planes.
Refinement. Refinement of *F*^2^ against ALL reflections. The weighted *R*-factor *wR* and goodness of fit *S* are based on *F*^2^, conventional *R*-factors *R* are based on *F*, with *F* set to zero for negative *F*^2^. The threshold expression of *F*^2^ > σ(*F*^2^) is used only for calculating *R*-factors(gt) *etc*. and is not relevant to the choice of reflections for refinement. *R*-factors based on *F*^2^ are statistically about twice as large as those based on *F*, and *R*- factors based on ALL data will be even larger.Part of the C_7_H_14_O_2_ molecule is disordered over two sites. In the refinement, the disordered carbon atoms were assigned common isotropic displacement parameters and geometric constraints.The dataset presented is one of a number of separate datasets collected from different crystals. Even so, the crystal diffracted very poorly, a fact reflected in the low percentage of observed structure factors, the use of common isotropic displacement parameters for the disordered parts, and the 'unreasonable' bond lengths.The *SORTAV* process was found to make no significant difference in this case which can be attributed to the Mo radiation and such a small crystal, the tiny mount and minimal oil, as one would expect. The *SORTAV* process was run as a check and to ensure that the equivalent reflections have been measured correctly - routine in our laboratory. The low percentage of observed reflections is due to the strenuous efforts which were made to measure everything possible with this poor crystal. The theta limit which was set for the data collection (27.5°) was probably higher than ideal but did ensure that nothing was missed. Again this was due to the very small, very poorly diffracting crystal.

### Fractional atomic coordinates and isotropic or equivalent isotropic displacement parameters (Å^2^)

**Table:**

	*x*	*y*	*z*	*U*_iso_*/*U*_eq_	Occ. (<1)
N1	0.25507 (7)	0.5059 (3)	0.03427 (7)	0.0424 (5)	
H1A	0.2774	0.6144	0.0409	0.051*	
H1B	0.2501	0.4962	0.0021	0.051*	
H1C	0.2689	0.3828	0.0455	0.051*	
C1	0.20393 (9)	0.5458 (5)	0.05763 (10)	0.0527 (7)	
H1D	0.1792	0.4317	0.0487	0.063*	
H1E	0.1893	0.6809	0.0456	0.063*	
C2	0.20856 (9)	0.5559 (4)	0.11088 (9)	0.0473 (7)	
C3	0.21988 (13)	0.7431 (5)	0.13377 (12)	0.0727 (9)	
H3A	0.2256	0.8670	0.1154	0.087*	
C4	0.22312 (17)	0.7544 (8)	0.18266 (15)	0.0993 (13)	
H4A	0.2314	0.8851	0.1977	0.119*	
C5	0.21472 (16)	0.5823 (9)	0.20932 (14)	0.0980 (13)	
H5A	0.2161	0.5924	0.2432	0.118*	
C6	0.20433 (14)	0.3944 (8)	0.18833 (14)	0.0905 (12)	
H6A	0.1992	0.2723	0.2074	0.109*	
C7	0.20111 (11)	0.3793 (5)	0.13874 (12)	0.0645 (8)	
H7A	0.1938	0.2468	0.1241	0.077*	
O1	0.33127 (6)	−0.1921 (3)	0.05299 (7)	0.0546 (5)	
O2	0.28197 (6)	0.0932 (3)	0.05844 (6)	0.0463 (5)	
C8	0.32360 (9)	−0.0055 (4)	0.06701 (8)	0.0418 (6)	
C9	0.36768 (10)	0.1019 (4)	0.09405 (10)	0.0534 (7)	
H9A	0.3868	−0.0068	0.1128	0.064*	
H9B	0.3924	0.1609	0.0705	0.064*	
C10	0.35192 (10)	0.2769 (5)	0.12763 (10)	0.0582 (8)	
H10A	0.3301	0.2162	0.1532	0.070*	
H10B	0.3302	0.3803	0.1098	0.070*	
C11	0.39793 (11)	0.3921 (5)	0.15034 (11)	0.0633 (8)	
H11A	0.4199	0.2876	0.1676	0.076*	
H11B	0.4195	0.4531	0.1246	0.076*	
C12	0.38396 (13)	0.5658 (5)	0.18440 (12)	0.0705 (9)	
H12A	0.3655	0.5034	0.2120	0.085*	
H12B	0.3594	0.6640	0.1682	0.085*	
C13	0.43051 (13)	0.6901 (6)	0.20273 (12)	0.0771 (10)	
H13A	0.4547	0.5922	0.2196	0.092*	
H13B	0.4494	0.7494	0.1751	0.092*	
C14	0.41686 (16)	0.8661 (7)	0.23573 (15)	0.1033 (13)	
H14A	0.4481	0.9514	0.2424	0.155*	
H14B	0.4036	0.8076	0.2657	0.155*	
H14C	0.3899	0.9551	0.2208	0.155*	
O3	0.41440 (7)	−0.3928 (3)	0.02418 (8)	0.0701 (6)	
H3	0.3877	−0.3358	0.0358	0.105*	
O4	0.35537 (7)	−0.5688 (3)	−0.01877 (8)	0.0704 (6)	
C15	0.40002 (10)	−0.5389 (5)	−0.00680 (11)	0.0555 (7)	
C16	0.44577 (11)	−0.6652 (6)	−0.02485 (12)	0.0709 (9)	
H16A	0.4710	−0.5666	−0.0395	0.085*	
H16B	0.4636	−0.7324	0.0028	0.085*	
C17	0.43247 (12)	−0.8326 (6)	−0.06032 (14)	0.0839 (11)	0.49
H17A	0.4200	−0.7641	−0.0901	0.101*	0.49
H17B	0.4033	−0.9183	−0.0478	0.101*	0.49
C18	0.4783 (3)	−0.9817 (12)	−0.0724 (2)	0.0717 (14)*	0.49
H18A	0.4777	−1.1102	−0.0522	0.086*	0.49
H18B	0.5124	−0.9096	−0.0680	0.086*	0.49
C19	0.4670 (3)	−1.0438 (12)	−0.1319 (3)	0.0877 (15)*	0.49
H19A	0.4450	−0.9367	−0.1483	0.105*	0.49
H19B	0.4999	−1.0645	−0.1496	0.105*	0.49
C20	0.4385 (3)	−1.2460 (14)	−0.1242 (3)	0.0993 (19)*	0.49
H20A	0.4595	−1.3307	−0.1013	0.119*	0.49
H20B	0.4053	−1.2108	−0.1082	0.119*	0.49
C21	0.4256 (5)	−1.3835 (17)	−0.1646 (4)	0.106 (2)*	0.49
H21A	0.4101	−1.5150	−0.1528	0.159*	0.49
H21B	0.4574	−1.4159	−0.1823	0.159*	0.49
H21C	0.4005	−1.3117	−0.1857	0.159*	0.49
C17'	0.43247 (12)	−0.8326 (6)	−0.06032 (14)	0.0839 (11)	0.51
H17C	0.4058	−0.7777	−0.0831	0.101*	0.51
H17D	0.4172	−0.9554	−0.0435	0.101*	0.51
C18'	0.4805 (3)	−0.9045 (10)	−0.0879 (3)	0.0717 (14)*	0.51
H18C	0.5128	−0.8886	−0.0684	0.086*	0.51
H18D	0.4842	−0.8238	−0.1179	0.086*	0.51
C19'	0.4673 (3)	−1.1640 (12)	−0.0990 (2)	0.0877 (15)*	0.51
H19C	0.4995	−1.2507	−0.1003	0.105*	0.51
H19D	0.4429	−1.2248	−0.0754	0.105*	0.51
C20'	0.4422 (3)	−1.1380 (14)	−0.1470 (3)	0.0993 (19)*	0.51
H20C	0.4650	−1.0449	−0.1662	0.119*	0.51
H20D	0.4087	−1.0625	−0.1426	0.119*	0.51
C21'	0.4310 (5)	−1.3384 (17)	−0.1765 (4)	0.106 (2)*	0.51
H21D	0.4203	−1.2983	−0.2090	0.159*	0.51
H21E	0.4028	−1.4191	−0.1615	0.159*	0.51
H21F	0.4627	−1.4255	−0.1777	0.159*	0.51

### Atomic displacement parameters (Å^2^)

**Table:**

	*U*^11^	*U*^22^	*U*^33^	*U*^12^	*U*^13^	*U*^23^
N1	0.0403 (11)	0.0341 (11)	0.0527 (12)	0.0018 (9)	−0.0060 (9)	−0.0029 (10)
C1	0.0403 (14)	0.0564 (17)	0.0615 (17)	0.0019 (12)	0.0005 (12)	−0.0009 (14)
C2	0.0397 (13)	0.0473 (16)	0.0549 (16)	0.0021 (11)	−0.0005 (12)	0.0002 (14)
C3	0.088 (2)	0.058 (2)	0.072 (2)	−0.0087 (17)	0.0125 (18)	−0.0083 (18)
C4	0.115 (3)	0.110 (3)	0.074 (3)	−0.019 (3)	0.005 (2)	−0.032 (3)
C5	0.090 (3)	0.145 (4)	0.059 (2)	0.008 (3)	0.000 (2)	−0.007 (3)
C6	0.083 (2)	0.113 (3)	0.077 (3)	0.031 (2)	0.017 (2)	0.043 (2)
C7	0.0605 (18)	0.0527 (18)	0.081 (2)	0.0095 (13)	0.0092 (16)	0.0057 (17)
O1	0.0454 (10)	0.0379 (11)	0.0804 (13)	0.0005 (8)	−0.0072 (9)	−0.0089 (10)
O2	0.0420 (10)	0.0388 (10)	0.0579 (11)	0.0010 (7)	−0.0100 (8)	−0.0012 (8)
C8	0.0416 (14)	0.0351 (14)	0.0485 (15)	−0.0015 (11)	−0.0029 (11)	0.0009 (12)
C9	0.0452 (14)	0.0510 (17)	0.0637 (17)	0.0000 (12)	−0.0121 (13)	−0.0049 (14)
C10	0.0517 (16)	0.0588 (18)	0.0637 (18)	−0.0013 (13)	−0.0142 (13)	−0.0090 (15)
C11	0.0556 (17)	0.0654 (19)	0.0686 (19)	−0.0050 (14)	−0.0186 (14)	−0.0105 (16)
C12	0.071 (2)	0.071 (2)	0.069 (2)	0.0005 (16)	−0.0136 (16)	−0.0146 (17)
C13	0.078 (2)	0.081 (2)	0.072 (2)	−0.0093 (18)	−0.0075 (17)	−0.0231 (19)
C14	0.106 (3)	0.101 (3)	0.103 (3)	−0.017 (2)	−0.001 (2)	−0.037 (2)
O3	0.0422 (11)	0.0786 (15)	0.0893 (15)	−0.0003 (9)	−0.0027 (10)	−0.0185 (12)
O4	0.0377 (11)	0.0696 (14)	0.1039 (17)	0.0009 (9)	−0.0006 (10)	−0.0212 (12)
C15	0.0392 (15)	0.0550 (18)	0.0724 (19)	−0.0002 (13)	0.0030 (14)	0.0057 (16)
C16	0.0455 (16)	0.087 (2)	0.080 (2)	0.0089 (15)	0.0068 (15)	0.0007 (19)
C17	0.0503 (18)	0.082 (2)	0.119 (3)	0.0050 (16)	0.0091 (18)	−0.026 (2)
C17'	0.0503 (18)	0.082 (2)	0.119 (3)	0.0050 (16)	0.0091 (18)	−0.026 (2)

### Geometric parameters (Å, º)

**Table:**

N1—C1	1.489 (3)	C14—H14B	0.9800
N1—H1A	0.9100	C14—H14C	0.9800
N1—H1B	0.9100	O3—C15	1.317 (3)
N1—H1C	0.9100	O3—H3	0.8400
C1—C2	1.495 (4)	O4—C15	1.200 (3)
C1—H1D	0.9900	C15—C16	1.508 (4)
C1—H1E	0.9900	C16—C17	1.488 (5)
C2—C3	1.375 (4)	C16—H16A	0.9900
C2—C7	1.377 (4)	C16—H16B	0.9900
C3—C4	1.372 (5)	C17—C18	1.544 (8)
C3—H3A	0.9500	C17—H17A	0.9900
C4—C5	1.338 (6)	C17—H17B	0.9900
C4—H4A	0.9500	C18—C19	1.732 (10)
C5—C6	1.351 (6)	C18—H18A	0.9900
C5—H5A	0.9500	C18—H18B	0.9900
C6—C7	1.393 (5)	C19—C20	1.489 (10)
C6—H6A	0.9500	C19—H19A	0.9900
C7—H7A	0.9500	C19—H19B	0.9900
O1—C8	1.260 (3)	C20—C21	1.461 (11)
O2—C8	1.254 (3)	C20—H20A	0.9900
C8—C9	1.511 (4)	C20—H20B	0.9900
C9—C10	1.510 (4)	C21—H21A	0.9800
C9—H9A	0.9900	C21—H21B	0.9800
C9—H9B	0.9900	C21—H21C	0.9800
C10—C11	1.517 (4)	C18'—C19'	1.704 (9)
C10—H10A	0.9900	C18'—H18C	0.9900
C10—H10B	0.9900	C18'—H18D	0.9900
C11—C12	1.500 (4)	C19'—C20'	1.489 (10)
C11—H11A	0.9900	C19'—H19C	0.9900
C11—H11B	0.9900	C19'—H19D	0.9900
C12—C13	1.511 (4)	C20'—C21'	1.538 (11)
C12—H12A	0.9900	C20'—H20C	0.9900
C12—H12B	0.9900	C20'—H20D	0.9900
C13—C14	1.491 (5)	C21'—H21D	0.9800
C13—H13A	0.9900	C21'—H21E	0.9800
C13—H13B	0.9900	C21'—H21F	0.9800
C14—H14A	0.9800		
			
C1—N1—H1A	109.5	H13A—C13—H13B	107.6
C1—N1—H1B	109.5	C13—C14—H14A	109.5
H1A—N1—H1B	109.5	C13—C14—H14B	109.5
C1—N1—H1C	109.5	H14A—C14—H14B	109.5
H1A—N1—H1C	109.5	C13—C14—H14C	109.5
H1B—N1—H1C	109.5	H14A—C14—H14C	109.5
N1—C1—C2	112.6 (2)	H14B—C14—H14C	109.5
N1—C1—H1D	109.1	C15—O3—H3	109.5
C2—C1—H1D	109.1	O4—C15—O3	123.5 (3)
N1—C1—H1E	109.1	O4—C15—C16	124.1 (3)
C2—C1—H1E	109.1	O3—C15—C16	112.4 (2)
H1D—C1—H1E	107.8	C17—C16—C15	115.4 (3)
C3—C2—C7	117.7 (3)	C17—C16—H16A	108.4
C3—C2—C1	121.0 (3)	C15—C16—H16A	108.4
C7—C2—C1	121.3 (3)	C17—C16—H16B	108.4
C4—C3—C2	121.3 (3)	C15—C16—H16B	108.4
C4—C3—H3A	119.3	H16A—C16—H16B	107.5
C2—C3—H3A	119.3	C16—C17—C18	114.4 (4)
C5—C4—C3	120.4 (4)	C16—C17—H17A	108.7
C5—C4—H4A	119.8	C18—C17—H17A	108.7
C3—C4—H4A	119.8	C16—C17—H17B	108.7
C4—C5—C6	120.3 (4)	C18—C17—H17B	108.7
C4—C5—H5A	119.8	H17A—C17—H17B	107.6
C6—C5—H5A	119.8	C17—C18—C19	103.4 (5)
C5—C6—C7	120.2 (4)	C17—C18—H18A	111.1
C5—C6—H6A	119.9	C19—C18—H18A	111.1
C7—C6—H6A	119.9	C17—C18—H18B	111.1
C2—C7—C6	120.1 (3)	C19—C18—H18B	111.1
C2—C7—H7A	120.0	H18A—C18—H18B	109.1
C6—C7—H7A	120.0	C20—C19—C18	97.6 (6)
O2—C8—O1	122.8 (2)	C20—C19—H19A	112.3
O2—C8—C9	119.9 (2)	C18—C19—H19A	112.3
O1—C8—C9	117.3 (2)	C20—C19—H19B	112.3
C10—C9—C8	116.0 (2)	C18—C19—H19B	112.3
C10—C9—H9A	108.3	H19A—C19—H19B	109.8
C8—C9—H9A	108.3	C21—C20—C19	120.4 (8)
C10—C9—H9B	108.3	C21—C20—H20A	107.2
C8—C9—H9B	108.3	C19—C20—H20A	107.2
H9A—C9—H9B	107.4	C21—C20—H20B	107.2
C9—C10—C11	113.7 (2)	C19—C20—H20B	107.2
C9—C10—H10A	108.8	H20A—C20—H20B	106.9
C11—C10—H10A	108.8	C19'—C18'—H18C	111.2
C9—C10—H10B	108.8	C19'—C18'—H18D	111.2
C11—C10—H10B	108.8	H18C—C18'—H18D	109.1
H10A—C10—H10B	107.7	C20'—C19'—C18'	98.1 (6)
C12—C11—C10	115.4 (2)	C20'—C19'—H19C	112.1
C12—C11—H11A	108.4	C18'—C19'—H19C	112.1
C10—C11—H11A	108.4	C20'—C19'—H19D	112.1
C12—C11—H11B	108.4	C18'—C19'—H19D	112.1
C10—C11—H11B	108.4	H19C—C19'—H19D	109.8
H11A—C11—H11B	107.5	C19'—C20'—C21'	117.9 (8)
C11—C12—C13	113.9 (3)	C19'—C20'—H20C	107.8
C11—C12—H12A	108.8	C21'—C20'—H20C	107.8
C13—C12—H12A	108.8	C19'—C20'—H20D	107.8
C11—C12—H12B	108.8	C21'—C20'—H20D	107.8
C13—C12—H12B	108.8	H20C—C20'—H20D	107.2
H12A—C12—H12B	107.7	C20'—C21'—H21D	109.5
C14—C13—C12	114.2 (3)	C20'—C21'—H21E	109.5
C14—C13—H13A	108.7	H21D—C21'—H21E	109.5
C12—C13—H13A	108.7	C20'—C21'—H21F	109.5
C14—C13—H13B	108.7	H21D—C21'—H21F	109.5
C12—C13—H13B	108.7	H21E—C21'—H21F	109.5
			
C1—C2—C3—C4	178.6 (3)	C8—C9—C10—C11	−174.7 (2)
C7—C2—C3—C4	−0.7 (5)	C9—C10—C11—C12	−179.4 (2)
C1—C2—C7—C6	−178.3 (3)	C10—C11—C12—C13	−174.6 (3)
C3—C2—C7—C6	1.0 (4)	C11—C12—C13—C14	178.7 (3)
C2—C3—C4—C5	−0.7 (6)	C16—C17—C18—C19	−146.8 (4)
C3—C4—C5—C6	1.8 (7)	C17—C18—C19—C20	−95.3 (6)
C4—C5—C6—C7	−1.5 (7)	C18—C19—C20—C21	−171.6 (8)
C5—C6—C7—C2	0.1 (6)		

### Hydrogen-bond geometry (Å, º)

**Table:**

*D*—H···*A*	*D*—H	H···*A*	*D*···*A*	*D*—H···*A*
N1—H1*A*···O1^i^	0.91	1.87	2.773 (3)	171
N1—H1*B*···O2^ii^	0.91	1.96	2.823 (3)	158
N1—H1*C*···O2	0.91	1.90	2.781 (3)	164
O3—H3···O1	0.84	1.78	2.610 (3)	172
C1—H1*D*···O4^iii^	0.99	2.60	3.542 (4)	160
C3—H3*A*···O2^i^	0.95	2.59	3.456 (4)	152

Symmetry codes: (i) *x*, *y*+1, *z*; (ii) −*x*+1/2, −*y*+1/2, −*z*; (iii) −*x*+1/2, −*y*−1/2, −*z*.
